# Planning CT Identifies Patients at Risk of High Prostate Intrafraction Motion

**DOI:** 10.3390/cancers15164103

**Published:** 2023-08-15

**Authors:** Hendrik Ballhausen, Minglun Li, Elia Lombardo, Guillaume Landry, Claus Belka

**Affiliations:** Department of Radiation Oncology, LMU University Hospital, LMU Munich, 81377 Munich, Germany

**Keywords:** radiation oncology, external beam radiotherapy, prostate carcinoma, intrafraction motion, motion management, risk management, planning CT

## Abstract

**Simple Summary:**

Motion of the prostate may adversely affect the outcome of radiotherapy. Online tracking of the prostate during irradiation is technologically feasible but only available at select institutions. It would be beneficial to be able to identify patients at risk of particularly high prostate intrafraction motion with simpler technology. In this paper, we present a larger inner diameter of the lesser pelvis as an anatomical predictor for high prostate intrafraction motion. It can be measured with a single planning CT, which should always be available. Risk patients identified in this way could then be selected for more rigorous online motion management or benefit from increased safety margins.

**Abstract:**

Prostate motion (standard deviation, range of motion, and diffusion coefficient) was calculated from 4D ultrasound data of 1791 fractions of radiation therapy in N = 100 patients. The inner diameter of the lesser pelvis was obtained from transversal slices through the pubic symphysis in planning CTs. On the lateral and craniocaudal axes, motility increases significantly (*t*-test, *p* < 0.005) with the inner diameter of the lesser pelvis. A diameter of >106 mm (ca. 6th decile) is a good predictor for high prostate intrafraction motion (ca. 9th decile). The corresponding area under the receiver operator curve (AUROC) is 80% in the lateral direction, 68% to 80% in the craniocaudal direction, and 62% to 70% in the vertical direction. On the lateral x-axis, the proposed test is 100% sensitive and has a 100% negative predictive value for all three characteristics (standard deviation, range of motion, and diffusion coefficient). On the craniocaudal z-axis, the proposed test is 79% to 100% sensitive and reaches 95% to 100% negative predictive value. On the vertical axis, the proposed test still delivers 98% negative predictive value but is not particularly sensitive. Overall, the proposed predictor is able to help identify patients at risk of high prostate motion based on a single planning CT.

## 1. Introduction

External beam radiotherapy (EBRT) is used in the treatment of prostate carcinoma [1,2,3]. The intrafraction motion of the prostate can significantly impact delivery to the prostate gland. The prostate gland is a mobile organ, and larger movements during treatment could result in irradiation of healthy tissue or underdosing of the tumor target volume, adversely affecting tumor control [4,5]. Conversely, patients with higher intrafraction motion might benefit from continuous tracking and intrabeam adjustments through smaller required safety margins [6,7].

Several studies have investigated the effects of intrafraction motion on EBRT of the prostate, and the results have demonstrated the need for motion management strategies to optimize treatment outcomes. For example, an early study found a maximal range of motion of 6.8 mm anterior and 4.6 mm posterior [8].

While intrafraction motion may be insignificant in one patient or fraction, it may be substantial in another. A study in 184 patients found a “large variation in typical shifts between” ranging from 1 to 6 mm radially [9].

Typically, the effect of prostate bed motion requires safety margins of 3 to 5 mm during image-guided radiation therapy [10].

In image guided therapy, several modalities have been available to pinpoint the location of the prostate and track its motion between fractions (“inter-fraction”). Examples include cone-beam computed tomography (CBCT), electronic portal imaging (EPI) with or without fiducial markers, stereotactic three-dimensional (3D) ultrasound and full 3D computed tomography [11,12]. The same or similar modalities are available to track the prostate’s motion during a fraction (“intra-fraction”).

One strategy is to limit intra-fraction motion by determining optimal levels of bladder filling [13] or restricting motion via endorectal balloons [14,15,16]. A more modern and generally advantageous approach is to reduce treatment times, limiting the opportunity for the prostate to wander off-beam [17].

Another approach is to use real-time tracking and beam adaptation, such as the Calypso 4D localization system, which allows for continuous monitoring and correction of the target position during treatment [18].

Similarly, four-dimensional (4D) ultrasound is a non-invasive technique used to visualize and track the motion of internal organs, including the prostate gland, during radiation therapy [19,20,21]. This method is not widely available in clinical practice, and its use is limited to specialized centers with the necessary equipment and expertise.

On the other hand, planning CT scans are routinely used in the treatment planning process for prostate cancer patients. This imaging modality provides high-quality images of the prostate gland and surrounding structures, which are used to generate a treatment plan that optimizes tumor coverage and minimizes the dose to nearby healthy tissues.

Because 4D ultrasound is not widely available, a planning CT would be particularly useful to identify patients at risk of high prostate intrafraction motion. In this paper, a CT-based anatomical criterion is derived and assessed. It may serve as a univariate predictor and identify patients at risk of high prostate intrafraction motion.

## 2. Materials and Methods

Infra-fraction motion of the prostate was recorded at our institution during 2.385 fractions of image-guided radiotherapy (IGRT) in 126 patients. The raw data is publicly available (see the data availability statement below) and has been described in detail at [22,23].

**Figure 1:**
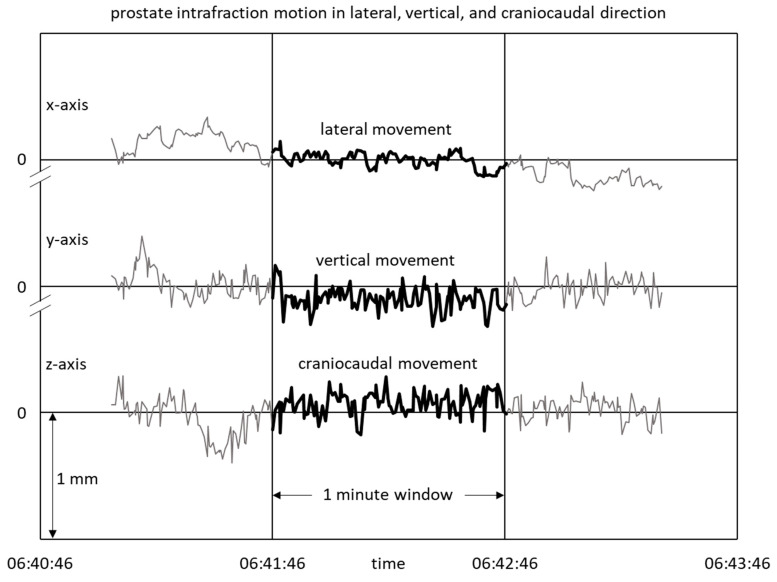
One minute of prostate tracking is evaluated per fraction.

For this paper, those fractions were selected for which ultrasound recordings of at least 2 min were available. Of each fraction, the central one-minute time window was selected for analysis; see Figure 1. The rationale was to work with recordings of standardized length and to exclude possible motion artefacts at the beginning or end of the recordings.

For each of the clipped recordings, the standard deviation *σ* of the prostate position, the range of motion *ρ*, and the diffusion coefficient *δ* of the random walk model [24,25] were calculated for each of the three axes. In the case of the lateral x-axis:

(1) $$\sigma_{x}=\sqrt{\frac{1}{N}\;\sum {\left(x_{i}-\bar{x}\right)}^{2}}$$

(2) $$\rho_{x}=\max\left(x\right)-\min\left(x\right)$$

(3) $$\delta_{x}=\frac{{\left(∆x\right)}^{2}}{∆T}$$

*σ* and *ρ* are measured in mm. To be comparable across fractions and patients, they require a static time window of fixed duration (here: one minute). The diffusion coefficient *δ* is measured in mm^2^ per minute and describes a linearly increasing variance over time.

Planning CTs were available for all patients. Transversal slices had been stored as DICOM images of 512 by 512 pixels with a pixel size of (1.074 mm)^2^ in 97 out of 102 cases, (1.367 mm)^2^ in 4 cases, and (1.073 mm)^2^ in 1 case. Using these pixel pitches, all measurements were converted to mm for further analysis.

**Figure 2:**
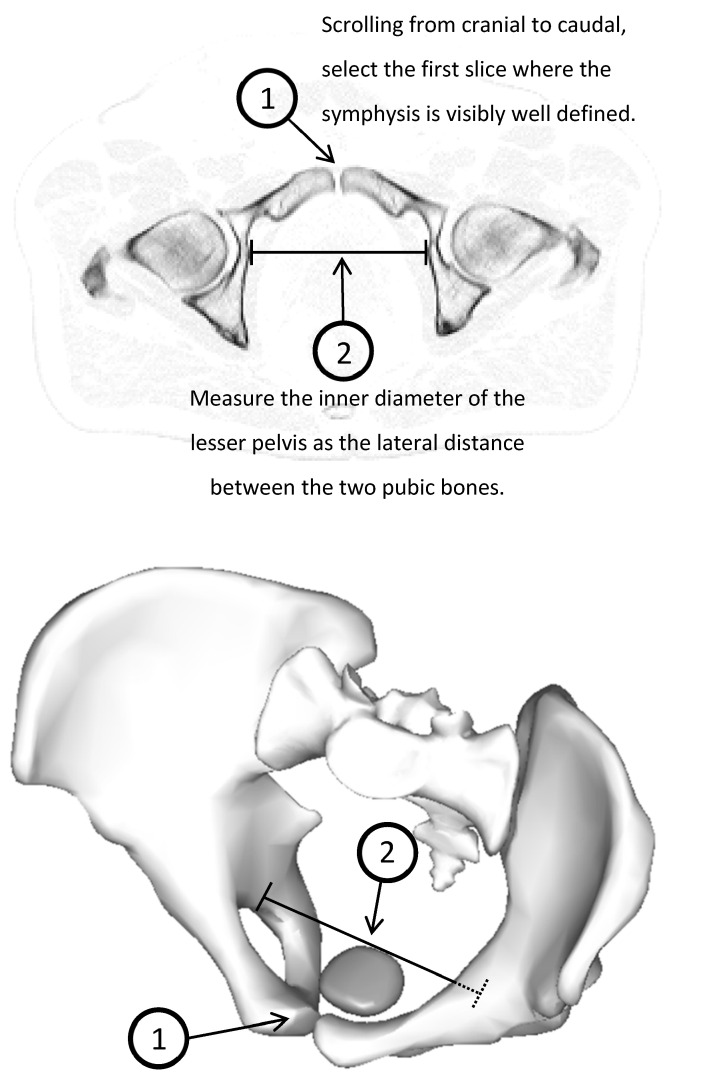
The inner diameter of the lesser pelvis is measured by identifying the most cranial slice of the planning CT that features the symphysis and then measuring the lateral distance between the two pubic bones. The 3D model shows the location of the prostate (1) in relation to the measured diameter (2) of the lesser pelvis.

Planning CTs were manually evaluated by a physicist. The inner diameter D of the lesser pelvis was measured; see Figure 2.

Raw data processing: For each patient, their inner diameter D of the lesser pelvis was tabulated together with their average standard deviation *σ*, range of motion *ρ*, and diffusion coefficient *δ* along each of the three axes (for a total of 10 data points per patient).

Calculation of aggregate statistics: Across all patients, the average ± standard deviation, the minimum, the median, the maximum, and the other two quartiles of the ten quantities were tabulated. Histograms of the ten quantities were plotted.

Exploratory statistics: Scatter plots of the patient-average standard deviation *σ*, range of motion *ρ*, and diffusion coefficient *δ* in relation to the inner diameter D of the lesser pelvis were drawn.

Receiver Operator Characteristics: Sensitivity against specificity was plotted, and the area under the receiver operator curve (AUROC) was measured.

Explaining variable: Any inner diameter of the lesser pelvis below the 6th decile is considered “low D”, while any diameter at or above the 6th decile is considered “high D”. The threshold was informed by receiver operator curves and selected to maximize sensitivity while still providing at least some specificity.

Graphical Analysis: Box plots of the patient-average standard deviation *σ*, range of motion *ρ*, and diffusion coefficient *δ* were drawn comparing patients with “low D” vs. “high D”.

Statistical Tests: Patient-average standard deviations *σ*, patient-average ranges of motion *ρ*, and patient-average diffusion coefficients *δ* above the 9th decile are considered “high”. The test predicts “high” prostate motility if and only if the inner diameter D of the lesser pelvis is “high”. Two-by-two contingency tables were drawn for each of the nine qualities, and the *p*-value was calculated by Fisher’s two-sided exact test. Sensitivity, specificity, negative predictive value (NPV), and positive predictive value (PPV) were calculated.

## 3. Results

### 3.1. Available Data

One patient had to be excluded from the analysis because their ultrasound recordings contained outlier data. Another patient had to be excluded from the analysis because the relevant transversal slice was not assessable because multiple metal implants cast shadows on the symphysis (“metal artefacts”). This reduced the number of available patients to N = 100 and the number of available fractions to 1791. Table 1 shows the format of the input data for all the following analyses.

### 3.2. Inner Diameter of the Lesser Pelvis

In the sample of N = 100 patients, the inner diameter D of the lesser pelvis ranged from 89 mm to 115 mm. The average diameter was 103 mm plus or minus 6 mm of standard deviation, and the median diameter was 104 mm. See Table 2. Note that these numbers are significantly lower than what is often reported as the transverse diameter of the pelvic inlet. The latter is commonly measured in females and in the superior pelvis.

**Figure 3:**
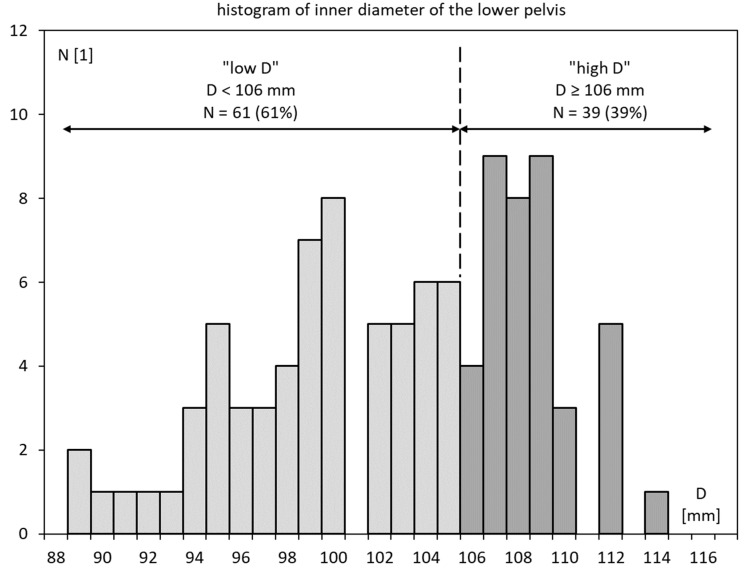
Histogram of the inner diameter of the lesser pelvis in the patient sample (D).

Figure 3 shows a histogram of D. In the following, D ≥ 106 mm is considered “high D” and D < 106 mm is labeled “low D”. Using this cutoff at the 6th decile, N = 61 or 61% of the patients were “low D”, and N = 39 or 39% were “high D”.

### 3.3. Prostate Motility

**Figure 4:**
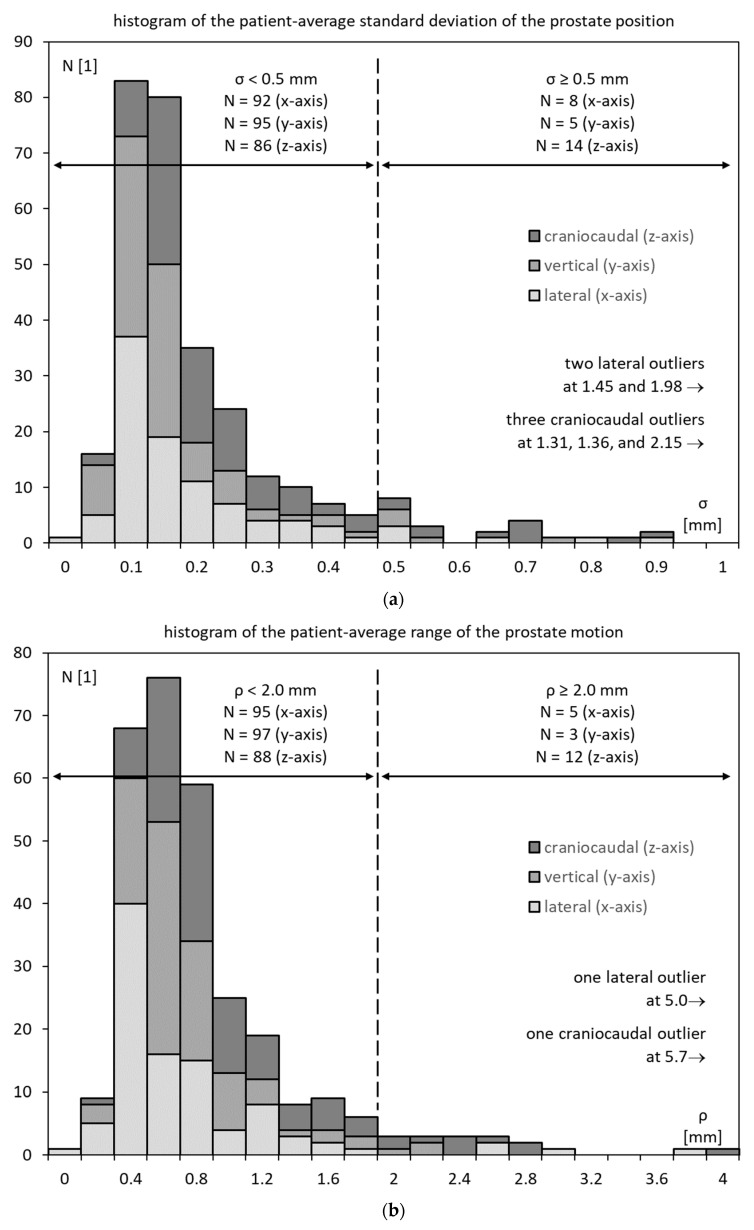

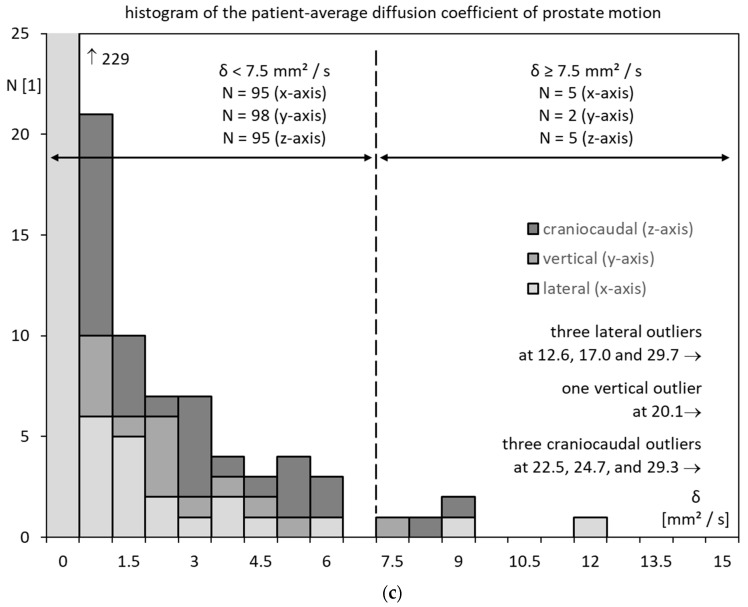
Histogram of patient-average (**a**) standard deviation *σ*; (**b**) range of motion *ρ*; (**c**) diffusion coefficient *δ*.

The patient-average standard deviation of the prostate position *σ* ranged from <0.1 mm to ca. 2.0 mm in the lateral and craniocaudal axes and 0.8 mm in the vertical axes. The average values were 0.25 mm (x-axis), 0.19 mm (y-axis), and 0.32 mm (z-axis), respectively. See Table 2 for further details. A joint histogram of all three axes is shown in Figure 4a. A joint cutoff of 0.5 mm corresponds roughly to the 9th decile.

Similarly, the patient-average range of motion of the prostate *ρ* ranged from <0.4 mm to ca. 2 mm on the y-axis and >5 mm on the x- and z-axes. The average values were 0.90 mm (x-axis), 0.84 mm (y-axis), and 1.22 mm (z-axis), respectively. See Table 2 for further details. A joint histogram of all three axes is shown in Figure 4b. A joint cutoff of 2.0 mm corresponds roughly to the 9th decile.

Finally, the patient-average diffusion coefficient of prostate motion ranged from close to zero to almost 30 mm^2^/s in some cases. High-motility cases pushed the averages to 1.46, 0.75, and 1.91 mm^2^/s in the three axes. However, medians were much more moderate at 0.20, 0.12, and 0.42 mm^2^, respectively. See Table 2 for further details. A joint histogram of all three axes is shown in Figure 4c. A joint cutoff of 7.5 mm is above the 9th decile, as the distribution is quite centered on zero.

In general, prostate motion was lower in the vertical direction than in the lateral and craniocaudal directions. This was true for all the tree measures *σ*, *ρ* and *δ*.

### 3.4. Prostate Motility vs. Inner Diameter of the Lesser Pelvis

**Figure 5:**
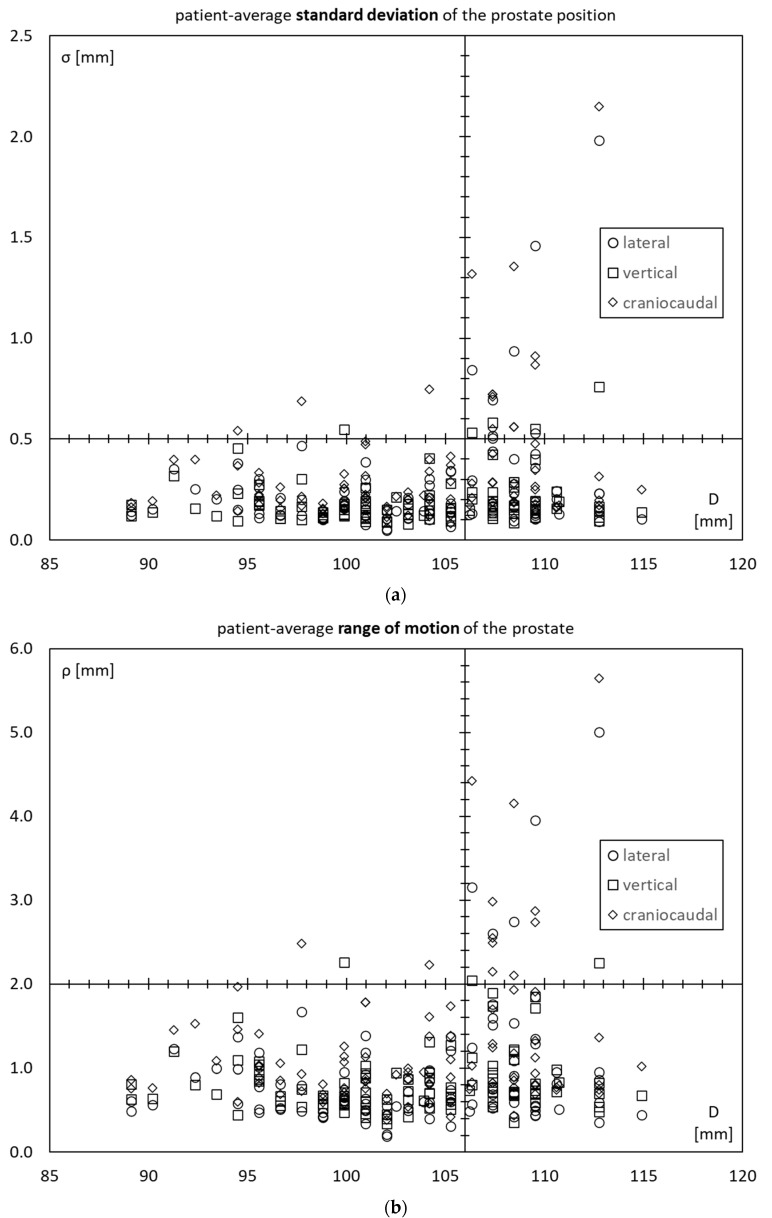

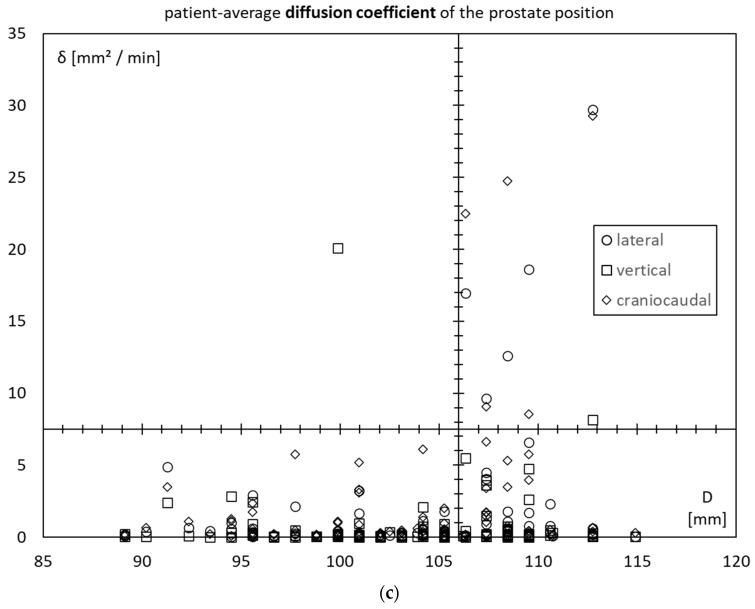
Scatter plots as a function of the inner diameter of the lesser pelvis (D) of the patient-average (**a**) standard deviation *σ*; (**b**) range of motion *ρ*; (**c**) diffusion coefficient *δ*.

The three scatter plots in Figure 5a–c show each 100 patients × 3 axes = 300 data points. The three plots show the patient-average standard deviation *σ*, patient-average range of motion *ρ*, and patient-average diffusion coefficient *δ*, respectively. The axes intersections are chosen such that they split each plot into four quadrants.

The lower two quadrants correspond to “low” prostate motility and contain most of the data points. There are a significant number of points both in the lower left and lower right quadrants. This means that there are a significant number of patients with low prostate motility, irrespective of the inner diameter of the lesser pelvis.

The upper two quadrants, however, contain significantly different points. These correspond to patients with “high” prostate motility. And most of them are found in the upper right quadrant, corresponding to high prostate motility and a high inner diameter of the lesser pelvis.

The picture is qualitatively similar for *σ*, *ρ*, and *δ*.

### 3.5. Receiver Operator Characteristics

**Figure 6:**
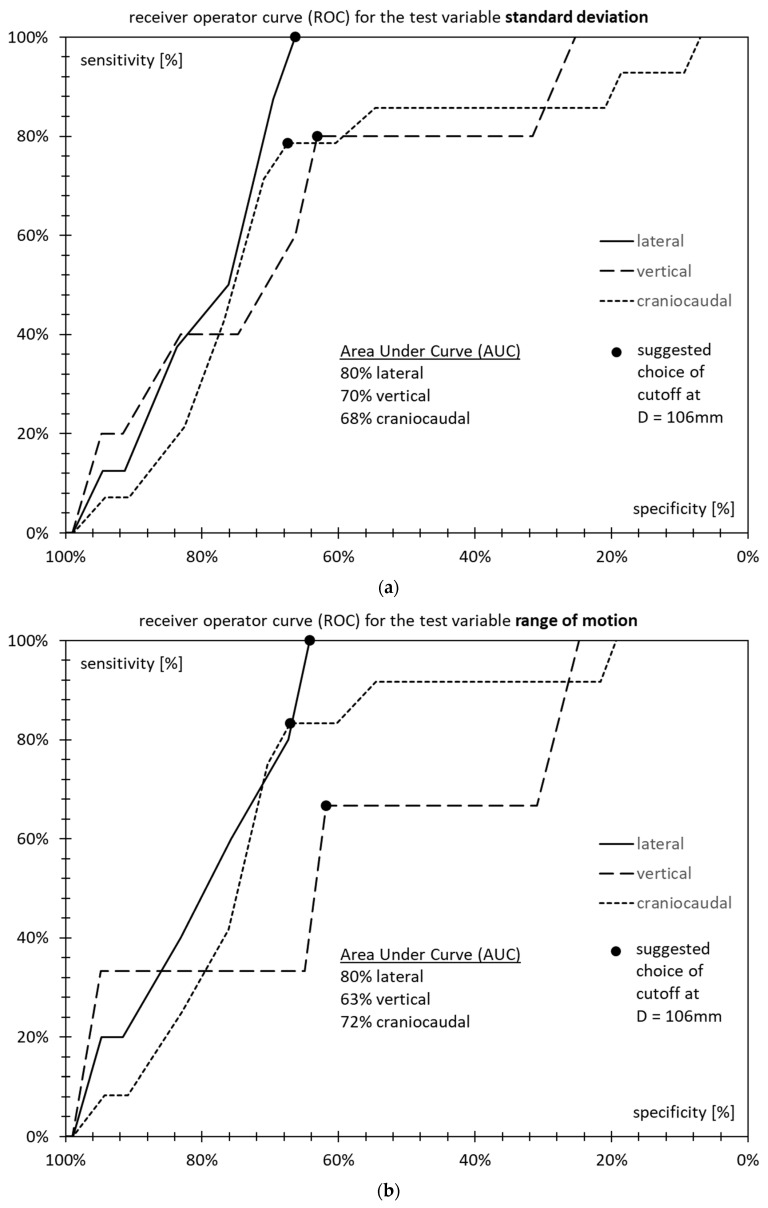

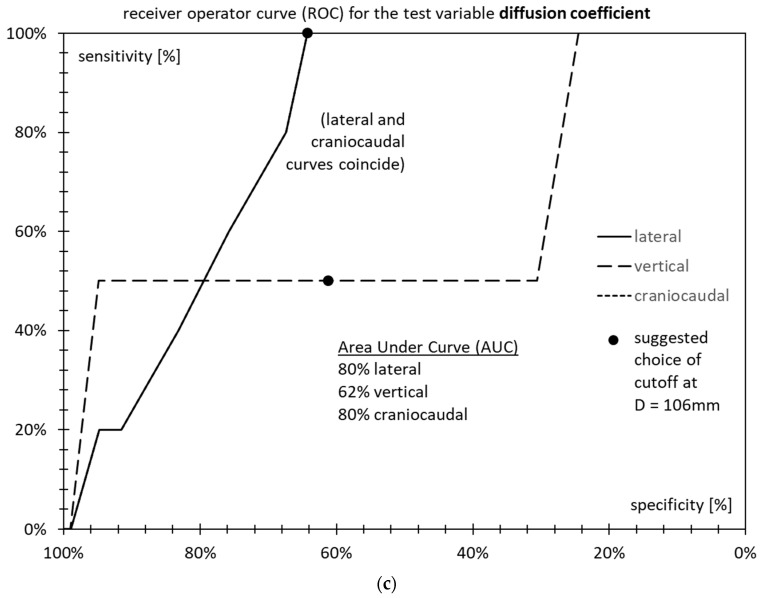
Receiver Operator Curves for the proposed test as a function of diameter D cutoff for (**a**) standard deviation *σ*; (**b**) range of motion *ρ*; (**c**) diffusion coefficient *δ*.

Figure 6a (standard deviation), Figure 6b (range of motion), and Figure 6c (diffusion coefficient) show receiver operator curves (ROC) for the suggested test. The tradeoff between sensitivity and specificity is a function of the choice of the cutoff diameter, D. The plots are shown for the patient-average standard deviation *σ*, patient-average range of motion *ρ*, and patient-average diffusion coefficient *δ*, respectively.

The area under the receiver operator curve (AUROC) is 80% in the lateral direction for all three motion characteristics. It is between 68% and 80% in the craniocaudal direction. In the vertical direction, it is only 62% to 70%.

Sensitivity is optimal for a choice of at most D = 106 mm and steeply falls off for higher choices of D, as most patients with high motility are in the range between 106 mm and 110 mm. Specificity, on the other hand, does not benefit from a lower choice of D, as even at high D, there are many patients that do not exhibit high motility.

At values of D lower than 106 mm, sensitivity does not increase anymore, but specificity only decreases further.

This is why D = 106 mm is chosen as the preferred cutoff for the following: As above, the regime D < 106 mm is called “low D” while anything at or above D ≥ 106 mm is considered “high D”.

### 3.6. Prostate Motility for Low D and High D

**Figure 7:**
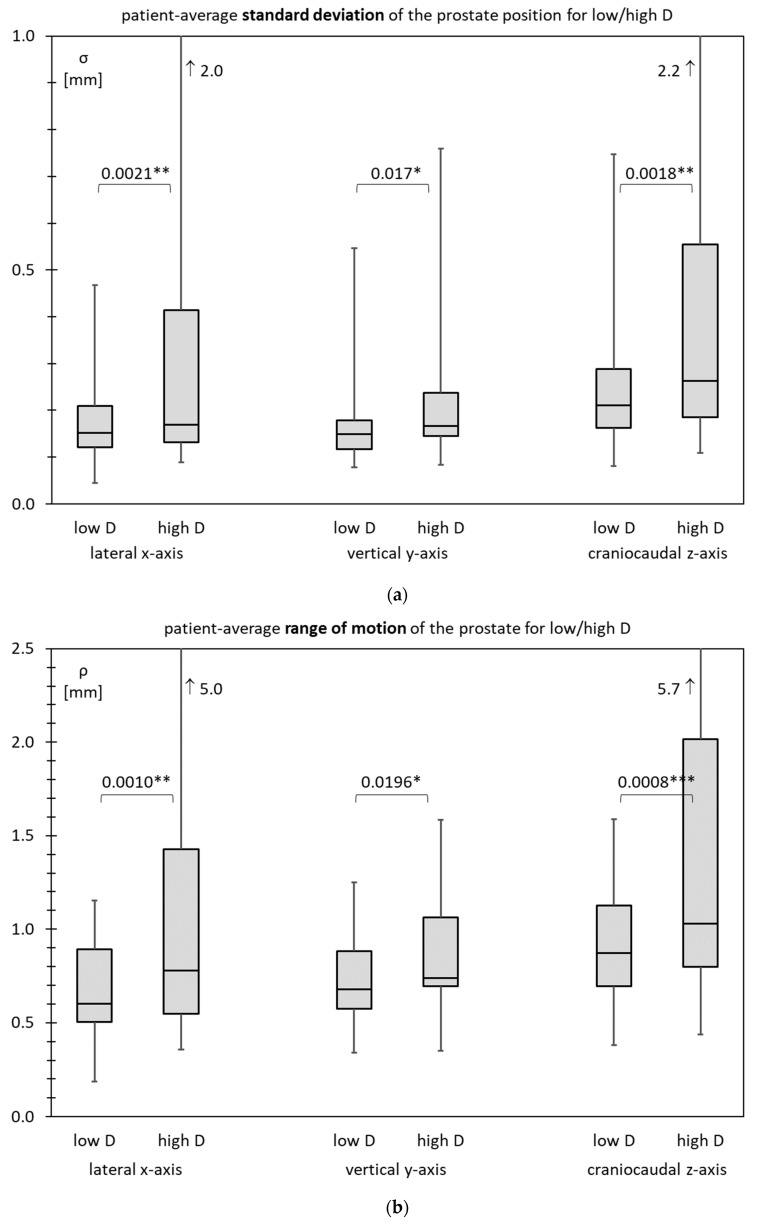

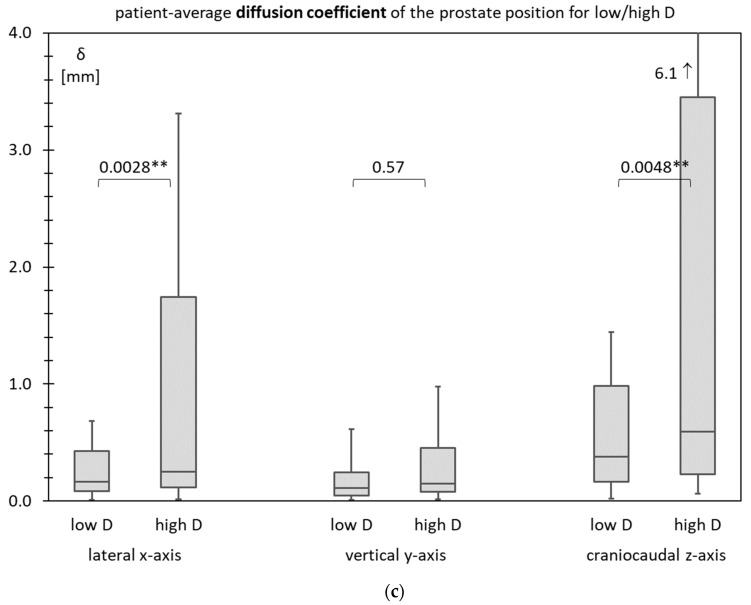
Box plots comparing low and high values of the inner diameter of the lesser pelvis (D) of the patient-average (**a**) standard deviation *σ*; (**b**) range of motion *ρ*; (**c**) diffusion coefficient *δ*. * *p* < 0.05, ** *p* < 0.01, *** *p* < 0.001.

Figure 7a–c show box plots for the patient-average standard deviation *σ*, patient-average range of motion *ρ*, and patient-average diffusion coefficient *δ*, respectively.

All prostate motility characteristics are visibly higher in cases of high D. The difference (by the unpaired *t*-test) is significant for all situations except for the diffusion coefficient on the vertical axis.

### 3.7. Test Statistics

Table 3a (standard deviation), Table 3b (range of motion), and Table 3c (diffusion coefficient) show two-by-two contingency tables for low/high D vs. low/high regimes of the prostate motility characteristics.

Fisher’s two-sided test fails for the vertical axis but is successful for both the lateral x-axis and the craniocaudal z-axis.

On the lateral x-axis, the proposed test is 100% sensitive and has a 100% negative predictive value for all three characteristics.

On the craniocaudal z-axis, the proposed test is 79% (standard deviation) resp. 83% (range of motion) resp. 100% (diffusion coefficient) sensitive and reaches 95% (standard deviation) resp. 97% (range of motion) resp. 100% (diffusion coefficient) negative predictive value.

On the vertical axis, the proposed test still delivers 98% negative predictive value but is not particularly sensitive.

In general, the proposed test shows little specificity, at only 61% to 67%. Generally, it only has little positive predictive value when <30%.

## 4. Discussion

Anatomical predictors of high prostate interfraction and intrafraction motion have been known before. Several studies have identified factors that are associated with increased prostate motion during radiation therapy, including the size and shape of the prostate gland and the presence of rectal and bladder filling.

For example, [26] found that “large bladder intrafractional filling and a large bladder volume difference from planning CT were more likely to experience bigger longitudinal prostate motion”. The study also derived an anatomical predictor where a smaller anterior–posterior size of the bladder and a smaller anterior–posterior to cranio–caudal ratio were favourable.

Perhaps closest in notion to our analysis, [27] uses the maximum rectal diameter (MRD) as a predictor for intrafraction prostate motion. They find that an MRD ≤ 3 cm predicts a prostate displacement ≤ 5 mm with 90% confidence.

Another calculational study derives a population model to estimate the probability of bladder presence during treatment using only the planning computed tomography [28] as in our study. Even earlier studies had already confirmed correlations between planning CT and intrafraction motion [29].

Our results are in line with the naive expectation that a larger prostate lodge and more leeway between the bony anatomy of the pelvis allow for higher prostate intrafraction motion.

In this study, only the center of gravity of the prostate (i.e., its location along the three spatial axes) was recorded by the instrument software. In follow-up work, we would also like to consider the size of the prostate (i.e., its volume) and other anatomical metrics to identify potential further confounding factors [30].

Independent analysis is needed to validate the criterion proposed in this paper in separate patients and unrelated datasets. Further research will be conducted into additional multivariate predictors in our dataset and into their validation by online MR Linac data.

## 5. Conclusions

An anatomical univariate predictor based on a single planning CT may help identify patients at risk of high prostate motion. While a diameter of the lesser pelvis of less than 106 mm has a high negative predictive value, patients with a larger diameter of the lesser pelvis may still exhibit low prostate motility. On the other hand, patients that are truly at risk are identified by D ≥ 106 mm with high sensitivity.

## Figures and Tables

**Table 1:** Sample of N = 100 patients of the inner diameter of the lesser pelvis (D) and patient-average prostate motion characteristics (*σ*, *ρ*, *δ*).

Patient	D [mm]	$\sigma_{x}$ [mm]	$\sigma_{y}$ [mm]	$\sigma_{z}$ [mm]	$\rho_{x}$ [mm]	$\rho_{y}$ [mm]	$\rho_{z}$ [mm]	$\delta_{x}$ [mm^2^/s]	$\delta_{y}$ [mm^2^/s]	$\delta_{z}$ [mm^2^/s]
1	102.0	0.143	0.086	0.155	0.549	0.455	0.633	0.181	0.034	0.223
2	99.9	0.169	0.125	0.148	0.615	0.579	0.661	0.084	0.181	0.099
3	97.7	0.467	0.300	0.688	1.668	1.219	2.484	2.140	0.493	5.739
4	109.5	0.427	0.154	0.866	1.344	0.694	2.869	1.688	0.067	5.759
5	109.5	0.103	0.110	0.178	0.444	0.596	0.810	0.057	0.013	0.132
…	…	…	…	…	…	…	…	…	…	…
100	101.0	0.300	0.181	0.487	1.183	0.887	1.782	1.678	0.098	3.107

**Table 2:** Distribution of the inner diameter of the lesser pelvis (D) and patient-average prostate motion characteristics (*σ*, *ρ*, *δ*).

N = 100	D [mm]	$\sigma_{x}$ [mm]	$\sigma_{y}$ [mm]	$\sigma_{z}$ [mm]	$\rho_{x}$ [mm]	$\rho_{y}$ [mm]	$\rho_{z}$ [mm]	$\delta_{x}$ [mm^2^/s]	$\delta_{y}$ [mm^2^/s]	$\delta_{z}$ [mm^2^/s]
average	103.3	0.25	0.19	0.32	0.90	0.84	1.22	1.46	0.75	1.91
std. dev.	5.9	0.26	0.12	0.29	0.72	0.39	0.85	4.16	2.32	4.59
minimum	89.1	0.05	0.08	0.08	0.19	0.34	0.38	0.01	0.01	0.02
1st quartile	99.9	0.12	0.12	0.17	0.51	0.61	0.76	0.09	0.05	0.18
median	104.2	0.16	0.16	0.22	0.65	0.71	0.89	0.20	0.12	0.42
3rd quartile	108.5	0.26	0.19	0.34	0.98	0.94	1.38	0.61	0.28	1.16
maximum	114.9	1.98	0.76	2.15	5.01	2.26	5.65	29.70	20.08	29.30

**Table 3:** (**a**) Contingency tables, significance, sensitivity, specificity, and negative/positive predictive value for the standard deviation. (**b**) Contingency tables, significance, sensitivity, specificity, and negative/positive predictive value for the range of motion. (**c**) Contingency tables, significance, sensitivity, specificity, and negative/positive predictive value for the diffusion coefficient.

Patient-Average Standard Deviation σ [mm]		Inner Diameter of the Lesser Pelvis D [mm]		Significance *p*-Value	Sensitivity Specificity	NPV PPV
		“Low D” < 106 mm	“High D” ≥ 106 mm			
(a)						
lateral (x-axis)	<0.5	0	8	0.0003	100%	100%
	≥0.5	61	31		66%	21%
vertical (y-axis)	<0.5	1	4	0.0743	80%	98%
	≥0.5	60	35		63%	10%
craniocaudal (z-axis)	<0.5	3	11	0.0020	79%	95%
	≥0.5	58	28		67%	28%
**Patient-Average Range of Motion *ρ* [mm]**		**Inner Diameter of the Lesser Pelvis** **D [mm]**		**Significance** ***p*-Value**	**Sensitivity** **Specificity**	**NPV** **PPV**
		**“Low D” < 106 mm**	**“High D” ≥106 mm**			
(**b**)						
lateral (x-axis)	<2.0	0	5	0.0076	100%	100%
	≥2.0	61	34		64%	13%
vertical (y-axis)	<2.0	1	2	0.5586	67%	98%
	≥2.0	60	37		62%	5%
craniocaudal (z-axis)	<2.0	2	10	0.0012	83%	97%
	≥2.0	59	29		67%	26%
**Patient-Average Diffusion** **Coefficient *δ* [mm^2^/s]**		**Inner Diameter of the Lesser Pelvis** **D [mm]**		**Significance** ***p*-Value**	**Sensitivity** **Specificity**	**NPV** **PPV**
		**“Low D” < 106 mm**	**“High D” ≥ 106 mm**			
(**c**)						
lateral (x-axis)	<7.5	0	5	0.0076	100%	100%
	≥7.5	61	34		64%	13%
vertical (y-axis)	<7.5	1	1	1.0000	50%	98%
	≥7.5	60	38		61%	3%
craniocaudal (z-axis)	<7.5	0	5	0.0076	100%	100%
	≥7.5	61	34		64%	13%

## Data Availability

Anonymous prostate motion datasets generated by 4D ultrasound are available in the Open Data LMU repository at https://data.ub.uni-muenchen.de/265 (accessed on 16 May 2022). A corresponding descriptor has been published at ref. [23]. Planning CT images are not publicly available due to data protection considerations. The lesser pelvis diameter, however, but no images, will be shared upon request with the corresponding author.
